# Next Generation Risk Assessment to Address Disease-Related Vulnerability—A Proof of Concept for the Sunscreen Octocrylene

**DOI:** 10.3390/toxics13020110

**Published:** 2025-01-29

**Authors:** María-Elena Fernández-Martín, Jose V. Tarazona

**Affiliations:** 1 ISCIII-UNEP PhD Programme on Biomedical Sciences and Public Health, Universidad Nacional de Educación a Distancia, 28040 Madrid, Spain; 2 Risk Assessment Unit, National Environmental Health Centre, Instituto de Salud Carlos III, Ministry of Science and Innovation, Carretera de Majadahonda a Pozuelo km 2.200, 28220 Madrid, Spain; jtarazona@isciii.es

**Keywords:** risk assessment, cosmetics, NGRA, octocrylene, UV filters, atopic dermatitis, oncological patient, endocrine disruptors

## Abstract

Risk assessment for cosmetics in the European Union (EU) are triggered by a ban on animal testing and concerns of endocrine disruption (ED). The risk assessment focuses on healthy populations and, for potential ED, includes specific developmental stages as vulnerable due to specific concerns on endocrine effects. However, the assessment focuses on healthy individuals and does not consider that some pathologies may increase dermal absorption and even vulnerability to endocrine disruptors. Data from the EU risk assessment, human pharmacokinetic studies and ToxCast bioactivity were combined in a hypothesis-driven Next-Generation Risk Assessment to identify possible risk drivers for vulnerable populations including oncological patients and atopic dermatitis. In vitro effects are observed at concentration in the order of measured plasmatic levels under normal use patterns. The induction of hepatic enzymes is the most relevant bioactivity endpoint, in line with animal findings. The information on endocrine potential is inconclusive, and the possibility for skin effects and endocrine mechanism linked to tumor induction require further elucidation. The information on octocrylene (CAS number: 6197-30-4) bioactivity is limited, lacking information on the metabolites and the immunotoxicity potential, particularly relevant for oncological patients.

## 1. Introduction

The market authorization of cosmetic ingredients differs among jurisdictions but, in general, includes the assessment of the potential risk of the cosmetic ingredients to the user under the expected use patterns. The cosmetic sector has been proactive regarding self-regulation [1]. The International Fragrance Association (IFRA) (www.ifrafragrance.org accessed on 14 May 2024), with the technical support of the Research Institute for Fragrance Materials (RIFM) (www.rifm.org accessed on 14 May 2024), establishes safety standards for fragrance ingredients, prohibiting or restricting the use of certain ingredients, which are compulsory for all IFRA members. RIFM has developed a safety assessment program with seven human health endpoints completed with environmental concerns [2] that currently includes over 250 standards. In addition, the EU and other jurisdictions have developed specific legislations regulating the scientific assessment of cosmetic ingredients and the marketing of the final product. A leading aspect of the EU legislation is the ban on the use of animal studies for assessing the safety of cosmetic ingredients. This has triggered specific efforts by the EU Scientific Committee on Consumer Safety (SCCS), Member State institutions and research organizations to develop risk assessment methodologies not requiring animal testing [3]. The assessment of dermal absorption and topical effects, including skin and eye irritation and sensitization, is facilitated through recent OECD (Organization for Economic Cooperation and Development) test guidelines based on alternative methods. If absorption is confirmed, the assessment of systemic toxicity using alternative methods to animal testing is still under development. New Approach Methodologies (NAMs) including high-throughput in vitro bioactivity method measuring are proposed as the basis for hypothesis-driven Next-Generation Risk Assessments (NGRAs) [4]. In addition, to avoid or at least minimize animal testing, NGRA approaches can also be used to address one of the key limitations of the current animal-based risk assessment paradigm, to extend the assessment from healthy populations to those with specific pathologies that could increase their vulnerability. The assessment of endocrine activity is an emerging issue, with increasing relevance in the area of cosmetics [5]. The susceptibility to endocrine disruptors (EDs) is particularly high during specific time windows of human development, including pregnancy and the fetal stage, childhood, puberty or menopause. In addition, diseases such as cancer or atopic dermatitis triggers concerns of the enhanced vulnerability to ED in cosmetics due to a combination of increased use/absorption and enhanced sensitivity to endocrine-mediated toxicity mechanisms [6].

The current chemical risk assessment paradigm focuses on setting acceptability toxicity thresholds for the “healthy” general population. Despite the scientific and legal efforts for minimizing and replacing the use of animal testing for assessing the safety of cosmetic ingredients, many cosmetic hazard assessments for systemic toxicity are still based on Points of Departure (PoDs) from animal studies [7]. The applicability of a PoD based on apical endpoints observed in healthy animals under controlled conditions should be considered with caution for assessing the risk to vulnerable human populations with specific diseases [8]. In fact, as the patients are under medical supervision, identifying the risk drivers for each vulnerable group and passing the information to the patient and the responsible medical services could be much more relevant than setting generic PoDs and acceptability threshold levels. Consequently, we propose the use of hypothesis-driven NGRA approaches, combining the available traditional assessments for the general population with other available information. The assessment includes endocrine-mediated effects as well as other relevant pathways. In particular, key elements from the toxicokinetic and toxicodynamic studies are combined with the available NAMs for identifying potential vulnerability drivers for the different groups of patients in order to improve their medical supervision.

A key element for this innovative approach is that the risk characterization does not put the focus on selecting acceptable thresholds but on identifying and communicating disease-related vulnerabilities that should trigger specific recommendations regarding the use of cosmetics containing ingredients that could lead to adverse effects in these vulnerable groups.

The UV filter octocrylene has been selected for this proof of concept due to the unresolved assessment on endocrine potential and its confirmed presence in marketed cosmetics intended for oncology patients and other vulnerable groups [6]. Octocrylene is an organic UV filter that absorbs mainly UVB radiation and UVA short wavelengths [9]. It is used in different cosmetic products to provide an adequate UV protection factor in sunscreen products or to protect cosmetic formulations from ultraviolet radiation. Systemic absorption of octocrylene after the application of sunscreens has been demonstrated [10,11]; 2-cyano-3,3-diphenylacrylic acid (CDAA) is the main metabolite, reaching higher concentrations than the parent in plasma and urine [10]. Octocrylene exposure in European citizens is increasing [12], and a detailed risk assessment by the SCCS was published in 2021 [13]. The SCCS assessment covered several cosmetic products containing 10% octocrylene, selected a dermal absorption value of 0.97 µg/cm^2^ and estimated systemic exposure levels up to 0.74 mg/kg bw per day for sunscreens and 0.84 mg/kg bw per day for aggregated exposure, including also octocrylene uses in lipsticks and creams [13]. Deriving a PoD from an extended one-generation reproductive toxicity study in rats, the SCCS concluded a safe use, although the assessment of endocrine disruption was inconclusive [13].

The use of sunscreens is a preventive measure against skin cancer [14]. Oncology patients require chronic and effective photoprotection due to the high incidence of skin cancer developing following cancer therapies. The EU risk assessment for the UV filter octocrylene was conducted by the SCCS using available animal oral toxicity studies for selecting the PoD for the hazard assessment [13]. The SCCS approach for the risk characterization is to estimate the Margin of Exposure (MoE) between the selected PoD and the estimated exposure levels, considering an acceptable risk level for the general population when the MoE is around 100 or higher.

## 2. Materials and Methods

The problem formulation focused on the risk for two vulnerable groups: oncological patients and those with atopic dermatitis. The hypothesis-driven conceptual model used information extracted from the EU assessment for the general “healthy” population of cosmetic users, conducted by the SCCS, as a background for selecting potential concerns and triggering the risk hypothesis.

In vitro bioactivity results for octocrylene were extracted from the USEPA CompTox Dashboard, using the Excel download option for further handling of the selected data. The assays with octocrylene activity were selected and assessed to identify mechanistic effects other than general cytotoxicity, endocrine-mediated effects and non-monotonic dose responses. These assessments were the basis for the identification of specific concerns regarding the vulnerability of subpopulation groups with specific diseases, focusing on oncological patients and those with atopic dermatitis.

For the hazard and exposure assessments, the data extracted from the references reported in the SCCS (2021) risk assessment [13] were complemented through a literature search in PubMed using “octocrylene” as the search term.

Exposure and hazard drivers were extracted independently from the SCCS assessment and from the ToxCast in vitro bioactivity and then integrated as specific vulnerability concerns for the selection of the most relevant risk hypothesis. When possible, the risk hypothesis was analyzed and implemented into evidence-based recommendations. The risk characterization method considered both deterministic and probabilistic approaches and was selected according to the available information for assessing each hypothesis. The evaluation was complemented with an uncertainty assessment and the identification of data gaps and testing needs.

In addition, the methodology developed by the EU project HBM4EU was used for proposing human health reference values for the general “healthy” population, and a discussion regarding possible adaptations for covering vulnerable groups. The methodology for deriving the HBM-GV_GenPop_ (human biomonitoring guidance value for general population) for octocrylene follows the option developed in the framework of the HBM4EU project [15] based on a defined external toxicity reference value, selecting the PoD in line with the final recommendations and the current EU approach [16,17]. Considering the toxicokinetic information, the selected biomarker was the CDAA (2-cyano-3,3-diphenylacrylic acid) metabolite.

The calculation considers the toxicity reference value (TVR); daily urinary excretion rate of the metabolite; molar corrections accounting, for the different molecular weights of parents and metabolites; and body weight-adjusted daily urine flow. See Formula (1) [15]:

(1) $${HBM-GV}_{GenPop}=\frac{TRV\times \frac{MW(Metabolite)\times Fue(Metabolite)}{MW(Substance)}}{Daily\;urinary\;flow\;rateadjusted\;to\;the\;bw}$$

HBM-GV_GenPop_ (human biomonitoring guidance value for the general population);

bw = body weight (kg);

TRV = toxicity reference value (mg/kg bw/d);

Daily urinary flow rate adjusted to the bw (ml/kg bw/d);

MW = molecular weight (g/mol);

Fue = molar urinary excretion factor.

## 3. Results and Discussion

### 3.1. Drivers and Identification of Risk Hypothesis

Two parallel assessments, one extracted from the SCCS (2021) opinion [13] and another from the ToxCast in vitro bioactivity (https://comptox.epa.gov/dashboard/chemical/invitrodb/DTXSID9025299 accessed on 16 January 2024) were conducted for the identification of risk drivers and indications of potential vulnerability for oncological and atopic dermatitis patients.

The main background hypothesis extracted from the SCCS assessment [13] are summarized in Table 1.

**Figure 1:**
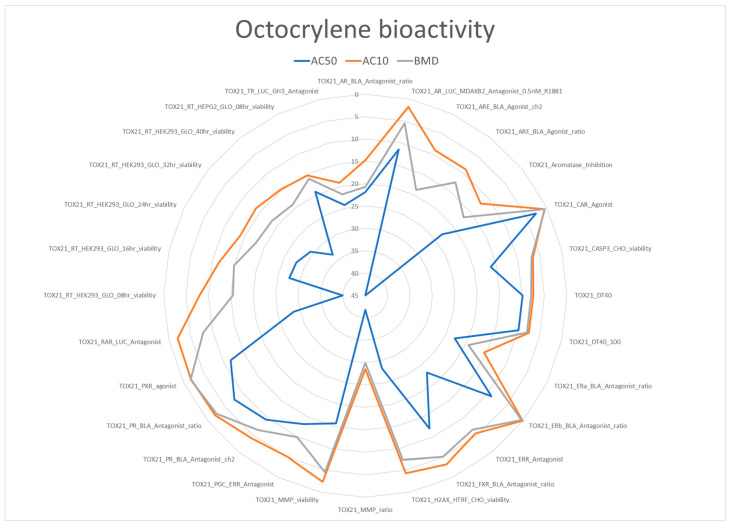
Summary of in vitro bioactivity extracted from ToxCast. All concentrations are in µM. The blue line represents the distribution of the AC50 (blue line—activity concentration at 50% of maximal activity) and is considered the most relevant information. AC10 (orange line—activity concentration at 10% of maximal activity) and BMD (grey line—Benchmark dose) are included for completeness.

The ToxCast database extraction included 218 bioactivity studies for octocrylene; bioactivity was observed in 28 studies, including 17 linked to specific genetic targets. Figure 1 summarizes the in vitro bioactivity of octocrylene. Most AC50s were around 5–20 µM; the most potent bioactivity was reported for TOX21_CAR_Agonist, 2.72 µM, followed by TOX21_PR_BLA_Antagonist_ratio, 7.62 µM. In addition to general (cito)toxicity, bioactivity drivers were observed for the four endocrine modalities EAST (estrogen, androgen, steroidogenesis and thyroid), although inconsistent and not triggering the ToxCast ER (estrogen receptor) and AR (androgen receptor) models. The activation of hepatic CYP3A4 (Cytochrome P450 3A4) linked to drug metabolism and the disruption of metabolism and energetic homeostasis were also reported. The bioactivity observations were used for identifying additional risk and vulnerability drivers, which are summarized in Table 2. Regarding bioactivity gaps, it should be noted that the available studies did not include those specific for assessing immunotoxicity and skin effects. Both are clear limitations regarding the identification of disease-related vulnerability drivers for the selected illnesses. In addition, the available data only covers the parent compound; no information on the bioactivity of the metabolite CDAA was found.

The interpretation of the in vitro ToxCast data requires the consideration of the “cytotoxicity burst” as many stress responses are activated in a nonspecific way at concentrations close to those resulting in cell death [18]. A cytotoxicity burst of 9.01 µM has been suggested for octocrylene [19]; three bioactivities are still below this value, suggesting that at least these three assays should be consider as specific activity linked to octocrylene mechanisms of action. One bioassay, TOX21 CAR Agonist, is linked to the activation of Cytochrome P450 with increased drug metabolism and also the implication on glucose and lipid metabolism; the other two are associated with nuclear receptors regulating the activity of transcription factors for the progesterone and estrogen receptors. Therefore, all risk and vulnerability drivers identified in Table 2 are confirmed by specific bioactivities observed at concentrations below the cytotoxicity burst.

The literature search identified 325 PubMed (accessed on 10 March 2024) documents published between 2010 and 2024; 63 mentioned octocrylene in the title. Following a screening by title and abstracts, five relevant publications were included in the analysis of the risk drivers. Several publications provided further confirmation regarding human exposure of susceptible groups and systemic distribution, detecting octocrylene in human mother milk [20] and their metabolites in urine [21,22]. The most relevant publication explored the use of ToxCast for assessing endocrine disruption potential [19].

### 3.2. Analysis of Risk Hypothesis, Vulnerabilities and Data Gaps

In line with the problem formulation, the hypothesis-driven assessment for the general population was followed by the identification and assessment of potential specific concerns for two vulnerable groups, oncological patients and those with atopic dermatitis.

SCCS exposure assessment follows the standard methodology for assessing external exposure, expressed as mg/kg bw per day, for different use patterns and routes. Dermal absorption was estimated as 0.97 µg/cm^2^ from an OECD TG 428 in vitro skin absorption (unpublished study report by Fabian and Landsiedel, 2020, summarized in SCCS (2021) [13]). Systemic absorption was confirmed in studies with healthy human volunteers; their averaged maximum plasmatic levels ranged from 0.6 to 11.7 µg/L (0.002 to 0.032 µM/L), covering different products and use patterns [10,11,23]. The additional literature search confirmed systemic distribution with a continuous increase in octocrylene exposure levels in the German general population since 1996 based on measured urinary CDAA levels up to 196 µg/L [12] and the presence of octocrylene in maternal milk, with measured values of 27.50 ± 22.15 g/g lipids [20].

The integration of use patterns and physicochemical properties revealed dermal accumulation and transfer to blood as the main exposure drivers for local and systemic assessments, respectively. The adaptation of the exposure drivers considered the potential for both specific use patterns and higher dermal absorption. The dermal distress of patients with atopic dermatitis is expected to increase application frequency [24] and absorption. The drivers for increased absorption are reduced physical barriers, increased and more superficial irrigation, and cell and hormone-mediated inflammatory responses. As octocrylene is lipophilic, both conditions may be associated not only with increased systemicity but also with increased accumulation potential. No information for proposing an absorption value for these patients could be retrieved, except the generic indication for drugs of an almost twofold increase compared to healthy individuals from a non-updated systematic review [25]. Human skin models reproducing atopic dermatitis have been developed for preclinical studies [26] and should be extended for studying the absorption of cosmetic ingredients intended for this vulnerable group.

For oncological patients, adaptation is less obvious and will depend on specific recommendations from physicians. Avoidance of sun exposure is a typical recommendation; if this is mainly achieved by physical barriers (hats and long-sleeve clothing), exposure will be reduced, while if the option is cosmetic protection, an increased frequency should be expected.

The adaptation of the toxicodynamic drivers considered the mechanisms associated with the assays with observed bioactivity and their potential links with enhanced vulnerability for the selected diseases. The SCCS evaluation and ToxCast bioactivity identified two common concerns, inconclusive potential for endocrine activity and clear evidence induction of CYP450 and other liver enzymes involved in hepatic metabolism. In addition, the ToxCast extraction suggested concerns regarding drivers for metabolism and energetic homeostasis, and lack of information on immunotoxicity and skin disruption drivers. As previously reported [19], the bioactivity AC_50_s are two orders of magnitude below the plasmatic levels measured in human volunteers, confirming low concerns for healthy individuals in line with the SCCS opinion. However, these generic assessments do not cover the disease-related vulnerabilities described in Table 2.

Endocrine activity is a vulnerability driver for oncological patients as several oncological processes are mediated by or associated with endocrine processes; the bioactivity is observed in some assays related to the four EAST modalities and require further assessment. Current approaches for identifying endocrine potential using ToxCast data focus on the combination of different assays measuring different elements of the EAST pathway to explore the overall agonistic or antagonistic activities; the estrogen [27] and androgen [28] models are well developed and included in regulatory guidance. However, in the case of disease-related vulnerabilities, a different approach is needed, as this vulnerability can be associated with a specific endocrine-related activity in a particular organ, tissue or cell type within a specific time window. Therefore, the assessment should focus on the relationship of the observed bioactivities with the pathogenesis of the disease and the possible consequences in terms of treatment and prognosis. Health status may also influence the dose–response slope; as a consequence, preventive measures should be considered even in the case of bioactivity observed at levels above those expected or measured in healthy individuals. The combination of the potential for increased skin absorption and the systemic bioactivity linked to increased drug metabolism and endocrine-related effects should be communicated to the professionals responsible for monitoring the health status of the vulnerable groups.

Regarding the identified data gaps, the three key areas mentioned in Table 2 require further investigation. Two main actions are suggested as a first step in an Integrated Approach to Testing and Assessment (IATA) focused on vulnerable groups: first action, to conduct in vitro studies with CDAA to address the bioactivity of this metabolite that reaches much higher plasmatic levels than the parent; second action, completing the high-throughput battery with the specific assays investigating immunotoxicity and effects on skin.

### 3.3. Estimation of Human Biomonitoring Guideline Values (HBM_GVs) for Octocrylene

The human biomonitoring guideline value for general population (HBM-GV _GenPop_) is a health-related guideline limit that refers to the internal body burden. It is derived for the general population and refers to the concentration of a substance or its metabolites in human biological material at which there is no risk of expected health effects during lifetime exposure [15]. Health-based HBM-GVs can be used directly to interpret HBM data and thus provide a better health risk assessment than a risk assessment based solely on external intake estimates. Health-based HBM-GVs have been developed for several substances within the HBM4EU Joint European Program [15,16] and various international bodies, but not yet for octocrylene.

Our estimation is based on the TRV derivation used as PoD the oral NOAEL (no observed adverse effect level) for octocrylene (0.765 mg/kg bw/day) proposed by the SCCS [13], divided by the standard uncertainty factor of 100 (10 intraspecific variability × 10 interspecific variability), and the proportional urinary excretion factor (Fue) of 0.45 (45%) [29]. Default daily urinary flow rates adjusted for body weight, 30 mL/kg bw/d and 20 mL/kg bw/d for children and adults, respectively, were used [15]; see Table 3.

The value for adults is three orders of magnitude higher than the average measured values in the German adult population based on human biomonitoring data in urine from the German population in winter [12] and 60 times higher than the average maximum value. However, higher HBM values are expected in situations of increased skin permeability due to altered skin barrier function and maximal exposure, such as during the summer, or throughout the year, as in the case of sun sensitive patients, such as oncological patients. The prospect and limitations regarding the use of HBM-GVs in risk assessment have been discussed previously [15,16]; the additional elements to be considered regarding their applicability to vulnerable groups are the possibility for modified metabolism, affecting the Fue obtained from healthy volunteers, and the extrapolation of a PoD from healthy animals to individuals with specific disease-related vulnerabilities.

The comparative results indicate little concern for the general population considering a standard risk assessment for the healthy population that does not specifically address risk related to endocrine disruption potential, confirming the need for a specific approach using an NGRA assessment to evaluate endocrine disruption and other concerns in vulnerable groups.

## 4. NGRA and Risk Management Options for Vulnerable Groups

**Figure 2:**
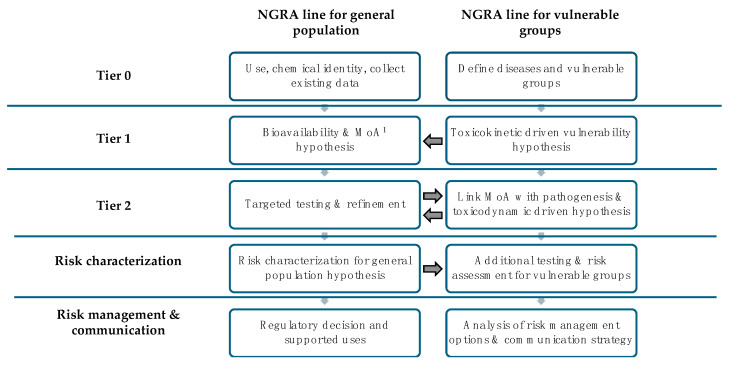
Proposed extension of the NGRA framework to cover disease-related vulnerabilities. The general population line should include susceptible groups (related to normal physiological development) such as children, pregnant women, adolescents undergoing puberty or elderly people. The parallel column focuses on vulnerabilities associated with pathological conditions including increased use; a column may be needed for each selected disease. ^1^ MoA: mode of action.

The current regulatory risk assessment practice for cosmetic ingredients focuses on healthy users; when used in products recommended for vulnerable groups, a complementary assessment should be conducted to refine the risk. This proof of concept for the UV filter octocrylene confirms the benefits of using hypothesis-driven NGRA for assessing the available information and identifying priorities for additional studies within an IATA strategy specifically formulated for addressing disease-related vulnerabilities. The SCCS Notes of Guidance, updated in 2023 [30], integrated the definition of NGRA as a human-relevant, exposure-led, hypothesis-driven risk assessment designed to prevent harm and the tiered framework proposed by Berggren et al. (2017) [31] adapted to cosmetics by Dent et al. (2018) [32]. The framework can be adapted to cover disease-related vulnerabilities through the incorporation of an additional column, as presented in Figure 2.

One additional element for assessments focusing on vulnerable groups is the consideration of the best risk management and risk communication options. The identification of concerns should be followed by specific indications, recommending vulnerable groups to not use the product or to follow specific measures to mitigate the risk. In addition, in line with the precautionary principle, the recommendations should be extended to inconclusive assessments. Depending on the concern, its severity and the uncertainties, another option for patients under health status control is to include a dedicated monitoring scheme of the potential consequences for the identified concerns, for example, to monitor the pharmacokinetics of anticancer drugs in oncological patients exposed to cosmetics enhancing hepatic metabolism. This approach could be the best option when the vulnerable group is small and well characterized, as well as when there are clear benefits related to the use of cosmetic products and there are no alternatives with confirmed low concerns. This requires an awareness campaign and specific efforts to pass the relevant information to the health professionals that are controlling the patients’ status.

## 5. Conclusions

Hypothesis-driven NGRA has been applied to assess the risk of the UV filter octocrylene as a cosmetic ingredient based on existing information as the first steps of the IATA strategy. Cosmetic products containing this ingredient are also recommended for population groups with specific pathologies, such as atopic dermatitis and oncological patients; therefore, a dedicated assessment for these vulnerable groups was included. No specific concerns have been identified for healthy individuals, confirming the SCCS conclusions. Following the assessment of vulnerability drivers, concerns have been identified related to increased absorption in patients with skin alterations. In addition, the inconclusive assessment regarding the potential for disrupting endocrine pathways and the confirmed capacity for increasing drug metabolism should be considered for oncological patients. The main data gaps for confirming vulnerability drivers are linked immunotoxicity and dermal alterations related to local accumulation following repeated use. An assessment fully based on in vitro methods would also require a parallel toxicological evaluation of the metabolite CDAA, which reaches much higher systemic concentrations than the parent under normal use patterns.

Following the analysis of this proof of concept, a proposal is presented for extending the NGRA framework to cover disease-related vulnerabilities. To avoid confusion, we have previously proposed to distinguish between susceptible and vulnerable groups; the first covers groups the general healthy population, with specific concerns linked to physiological conditions due to developmental status (fetus, children, adolescents undergoing puberty, women who are experiencing pregnancy and elderly people), and the second is linked to persons with specific diseases that may increase the exposure and/or effects of the ingredient exposure and which currently are not covered in the standard regulatory risk assessments.

## Figures and Tables

**Table 1:** Summary of risk hypothesis and vulnerability concerns extracted from the SCCS (2021) assessment [13].

Observation/Conclusion	Relevance	Risk and Vulnerability Drivers
Cutaneous absorption confirmed	Need to address systemic effects	Absorption may be higher in patients with skin disorders
Inhalation exposure is significant for propellant sprays	High variability in inhalation exposure	Selection of pump vs. propellant sprays reduces ca. 100 times inhalation exposure
Rapid metabolism, plasmatic levels of metabolite CDAA ^1^ significantly higher than the parent compound	Systemic toxicity may be related to the parent ingredient, the metabolite(s) or both	Bioactivity data should cover both the parent ingredient and the relevant metabolite(s)
CDAA represents the larger fraction of renal excretion	CDAA urinary levels are the best marker for human biomonitoring	Reference values for human biomonitoring could be established for the general population
There are some but inconclusive indications of endocrine activity	All modalities associated with endocrine activity to be considered	Potential for endocrine disruption vulnerabilities
Induction of hepatic enzymes leading to increased metabolism of thyroid hormones	The thyroid disruption is assumed to be rat-specific and not relevant for humans	Vulnerabilities associated with increased hepatic metabolism
PoD for systemic toxicity 76.5 mg/kg bw per day, based on an oral rat reproductive NOAEL ^2^ and 50% absorption	Reproductive NOAEL low relevance for disease-related vulnerability	Standard hazard assessment for the general “healthy” population
Plasmatic levels in studies with human volunteers	Expected exposure levels under normal use patterns	Baseline exposure for toxicodynamic-based vulnerability

^1^ CDAA: 2-cyano-3,3-diphenylacrylic acid; ^2^ NOAEL: no observed adverse effect level.

**Table 2:** Summary of risk hypothesis and vulnerability concerns extracted from the ToxCast in vitro bioactivity studies.

Bioactivity Pathway	Relevance	Risk and Vulnerability Drivers
Inconsistent/inconclusive assessment of EAST ^1^ endocrine modalities	The results are insufficient for the identification of octocrylene as an endocrine disrupter, but bioactivity was detected	Potential for endocrine disruption vulnerabilities
PXR ^2^ agonist and induction of Cytochrome P450 including Cytochrome P450 3A4 (CYP3A4)	Induction of hepatic drug metabolisms; also involved in the metabolisms of hormones and other substances	Driver for oncology patients, as may affect the efficacy of patients’ treatments
FXR ^3^ and ERR ^4^ antagonist affecting genes regulating metabolisms and cellular energetic pathways	May affect energy homeostasis; bioactivity shared with several anticancer drugs	Potential for metabolic disruption; driver for oncology patients due to potential for interaction with anticancer drugs
No information on bioactivity of the metabolite(s)	CDAA plasmatic levels are much higher, and the in vivo studies do not clarify if the metabolite plays a role in the toxicity	Driver for generating additional information: bioactivity of CDAA
No information on skin-related pathways	Very relevant for cosmetic uses in general and specifically for patients with atopic dermatitis	Driver for generating additional information to assess vulnerability for patients with dermal disruption
No information on immunotoxicity pathways	Very relevant for oncological patients	Driver for generating additional information to assess vulnerability for oncological patients

^1^ EAST: estrogen, androgen, steroidogenesis and thyroid; ^2^ PXR: pregnane X receptor; ^3^ FXR: farnesoid X receptor; ^4^ ERR: estrogen-related receptor; ^5^ CDAA: 2-cyano-3,3-diphenylacrylic acid.

**Table 3:** Proposed HBM-GVs for octocrylene, and parameters used for the derivation.

HBM-GV _GenPop_			TRV	MW Metabolite CDAA	MW Octocrylene	Fue	Daily Urinary Flow Rate Adjusted to bw
adults	11.87	mg/L	0.765	249.27	361.5	0.45	20
children	7.91	mg/L	0.765	249.27	361.5	0.45	30

HBM-GV_GenPop_ (HBM-GV derived for the general population); TRV: toxicity reference value (mg/kg bw/d); MW: molecular weight (g/mol); Fue: molar urinary excretion factor; daily urinary flow rate adjusted to the bw (ml/kg bw per day); bw: body weight (kg).

## Data Availability

All data supporting the reported results are publicly available and can be found in the references and websites indicated in through the document.
